# Supplementary material for: Antiparasitic Drugs against SARS-CoV-2: A Comprehensive Literature Survey
Source: Microorganisms. 2022 Jun 24;10(7):1284. doi: 10.3390/microorganisms10071284 (PMC9320270; doi:10.3390/microorganisms10071284)
Supplement: Supplementary file 1 [file microorganisms-10-01284-s001.zip › microorganisms-1736203-supplementary.pdf]

microorganisms

Supplementary Table S1. CQ and/or HCQ clinical trials without available results.

Chloroquine

Trial No.	Phase	Drugs	No. participants	Status
NCT04342650	Phase 2	CQ diphosphate vs Placebo oral tablet	152	Completed, no results
NCT04351295	Phase 2 / 3	CQ vs favipiravir	92	Completed, no results
NCT04328493	Phase 2	CQ phosphate	10	Completed, no results
NCT04627467	Phase 2	CQ	3217	Completed, no results
NCT04331600	Phase 4	CQ phosphate vs Telemedicine	16	Completed, no results
NCT04353336	Phase 2 / 3	CQ	194	Completed, no results
NCT04709744	N/A	CQ pretreatment and Vitamin levels and supplement on systemic lupus erythematosus (SLE) and COVID-19 patients	38	Completed, no results
NCT04333628	Phase 2 / 3	CQ vs SoC	5	Terminated
NCT04344951	Phase 2	CQ phosphate (200 mg tablets)	29	Terminated
NCT04443270	Phase 1	CQ phosphate	200	Not yet recruiting

Hydroxychloroquine

Trial No.	Phase	Drugs	No. participants	Status
NCT04391127	Phase 3	HCQ vs IVM vs Placebo	108	Completed, no results
NCT04384380	N/A	HCQ Sulfate 200 MG [Plaquenil]	33	Completed, no results
NCT04788355	Phase 3	Control group (standard hospital treatment) vs HCQ vs HCQ+apixaban vs Apixaban	176	Completed, no results
NCT04321278	Phase 3	HCQ vs HCQ+Azithromycin	440	Completed, no results
NCT04434144	N/A	HCQ+Azithromycin vs IVM+Doxycycline	116	Completed, results submitted but not available
NCT04481633	N/A	HCQ. Protective effect of pretreatment with HCQ in patients with lupus (SLE) or Gougerot's disease (SGD).	800	Recruiting
NCT04471649	N/A	HCQ in COVID-19 patients with rheumatoid arthritis	80	Recruiting
NCT04481633	N/A	HCQ +/- immunosuppressants	800	Recruiting
NCT04964583	Phase 2 / 3	HCQ+Azithromycin	105	Recruiting

NCT04352933	Phase 3	HCQ daily or weekly dosing vs Placebo (prophylactic treatment for frontline healthcare workers)	1000	Recruiting
NCT04316377	Phase 4	HCQ sulphate	53	Active, not recruiting
NCT04860284	Phase 2	HCQ tablets for the treatment of non-severe COVID-19	105	Active, not recruiting
NCT04374903	N/A	HCQ+AZ vs HCQ+SIR (Sirolimus)	58	Not yet recruiting
NCT04443725	Phase 2 / 3	HCQ, Sofosbuvir, daclatasvir vs SoC	100	Not yet recruiting

Chloroquine and/or Hydroxychloroquine

**Trial No.**	**Phase**	**Drugs**	**No. participants**	**Status**
NCT04447534	Phase 3	CQ or HCQ + Zinc supplementation (enhance of clinical efficacy)	191	Completed, no results
NCT04420247	Phase 3	CQ or HCQ vs SoC	142	Completed, no results
NCT04362332	Phase 4	CQ Sulfate vs HCQ vs SoC	25	Terminated
NCT04351191	Phase 4	HCQ Sulfate Regular dose vs HCQ Sulfate Loading Dose vs CQ vs Placebo	137	Terminated
NCT04346667	Phase 4	HCQ Sulfate Regular dose Vs HCQ Sulfate Loading Dose Vs CQ vs Placebo	125	Terminated
NCT04351724	Phase 2 / 3	Study: CQ or HCQ, lopinavir/ritonavir, remdesivir or SoC.<br>Substudy A: rivaroxaban vs SoC<br>Substudy B: renin-angiotensin (RAS) blockade vs no RAS blockade<br>Substudy C: asunercept / pentglobin vs SoC	500	Recruiting
NCT04351724	Phase 2 / 3	Study: CQ or HCQ, lopinavir/ritonavir, remdesivir or SoC.<br>Substudy A: rivaroxaban vs SoC<br>Substudy B: renin-angiotensin (RAS) blockade vs no RAS blockade<br>Substudy C: asunercept / pentglobin vs SoC	500	Recruiting
NCT04303507	N/A	CQ or HCQ vs Placebo	40000	Active, not recruiting
NCT04303507	N/A	CQ or HCQ vs Placebo	40000	Active, not recruiting
NCT04393051	Phase 2	CQ / HCQ + low-molecular weight heparin (LMWH) with or without baricitinib as adjunctive therapy	126	Not yet recruiting
NCT04393051	Phase 2	CQ / HCQ + low-molecular weight heparin (LMWH) with or without baricitinib as adjunctive therapy	126	Not yet recruiting

Supplementary Table S2. Clinical trials on the use of Artemisinin and derivatives without available results.

Trial No.	Phase	Drugs	No. participants	Status
NCT04382040	Phase 2	ArtemiC or CimetrA (Artemisinin, Curcumin, Frankicense and Vitamin C)	50	Completed, no results
NCT04802382	Phase 3		252	Recruiting
NCT04801017	Phase 2	Artemisinin +/- OT-101 + SoC	18	Not yet recruiting
NCT04387240	Phase 2	Artesunate	22	Not yet recruiting
NCT05273242	Phase 4	Artesunate +/- SoC	400	Recruiting
NCT04475107	Phase 2	Pyramax (Pyronaridine - Artesunate)	113	No results
NCT05084911	Phase 3	Pyramax (Pyronaridine - Artesunate)	1420	Recruiting
NCT04532931	Phase 2	Pyronaridine - Artesunate<br>Artesunate - Amodiaquine	192	No results
NCT04701606	Phase 2 / 3	Artecom® (Pyronaridine - Artesunate)	402	Recruiting
NCT04695197	Phase 3	Pyronaridine - Artesunate<br>Artemether - Lumefantrine	142	Recruiting
NCT04502342	Phase 2	Cospherunate (Artesunate - Amodiaquine) + Azythromycine +/- Phytomedicine	30	Enrolling by invitation

Supplementary Table S3. Clinical trials involving Niclosamide as antiviral agent for COVID-19 patients' treatment without published results.

Trial No.	Phase	Drugs	No. participants	Status
NCT04749173	Phase 1	Injectable Niclosamide	24	Completed, no results
NCT04750759	Phase 2	Niclosamide+Camostat<br>Placebo	4	Completed, no results
NCT04541485	Phase 1	Injectable Niclosamide<br>Placebo	2	Completed, no results
NCT04753619	Phase 2	Niclosamide oral tablets	150	Recruiting
NCT04558021	Phase 3	Niclosamide suspension<br>Placebo	200	Recruiting
NCT04603924	Phase 2 / 3	Niclosamide / Placebo	436	Recruiting
NCT04870333	Phase 2 / 3	Niclosamide / Placebo	1500	Recruiting
NCT05087381	Phase 4	Niclosamide / Other drugs	1800	Recruiting

NCT04932915	Phase 2	Niclosamide / Placebo	330	Terminated
NCT04542434	Phase 2	Niclosamide oral tablets / Placebo	148	Withdrawn
NCT04436458	Phase 2	Niclosamide oral tablets / Placebo	100	Withdrawn
NCT05226533	Phase 2	Injectable Niclosamide / Placebo	60	Not yet recruiting
NCT04858425	Phase 2	Niclosamide oral tablets / Placebo	166	Active, not recruiting

Supplementary Table S4. IVM clinical studies without results posted and/or published.

Trial No.	Phase	Drugs	No. participants	Status
NCT04646109	Phase 3	IVM	66	Completed, no results
NCT04920942	Phase 3	IVM	500	Completed, no results
NCT04739410	Phase 4	IVM	50	Completed, no results
NCT04894721	Phase 2, 3	IVM vs Placebo	172	Completed, no results
NCT04668469	N/A	IVM vs HCQ	600	Completed, no results
NCT04407130	Phase 2	IVM+Doxycycline+Placebo vs IVM+Placebo vs Placebo	72	Completed, no results
NCT04746365	Phase 4	IVM vs HCQ vs Placebo	300	Completed, no results
NCT04673214	Phase 3	IVM+AZ+Ribaroxaban+Paracetamol vs AZ+Ribaroxaban+Paracetamol	114	Completed, no results
NCT04403555	Phase 2, 3	IVM	164	Completed, no results
NCT05076253	Phase 1, 2	IVM vs Placebo	72	Completed, no results
NCT04407507	Phase 2	IVM vs Placebo	66	Completed, no results
NCT04635943	Phase 2	IVM vs Placebo	186	Completed, no results
NCT04632706	Phase 1	IVM vs Placebo	24	Completed, no results
NCT04530474	Phase 3	IVM vs Placebo	0	Withdrawn
NCT04891250	Phase 4	IVM vs Prophylaxis	0	Withdrawn
NCT04703205	Phase 2	IVM vs Placebo	214	Active, not recruiting
NCT05040724	Phase 3	IVM	200	Active, not recruiting
NCT05155527	Phase 2	IVM Tablets vs Placebo	200	Not yet recruiting

NCT04360356	Phase 2,3	IVM plus Nitazoxanide vs Soc	100	Not yet recruiting
NCT05060666	Phase 3	IVM vs Placebo	412	Not yet recruiting
NCT04510233	Phase 2	IVM nasal vs IVM oral vs SoC	60	Not yet recruiting
NCT04937569	Phase 3	IVM Tablets	1644	Not yet recruiting
NCT04712279	Phase 2,3	IVM 0.6mg/kg/day vs IVM 1.0mg/kg/day vs HCQ vs Placebo	294	Not yet recruiting
NCT04886362	Phase 2,3	IVM vs Placebo	966	Not yet recruiting
NCT04944082	Phase 4	IVM+Remdesivir	60	Not yet recruiting
NCT04527211	Phase 3	IVM	550	Not yet recruiting
NCT04551755	Phase 2	IVM+Doxycycline vs Placebo	188	Not yet recruiting
NCT04768179	Phase 2, 3	IVM+Aspirin+SoC	490	Not yet recruiting
NCT04392427	Phase 3	IVM+Nitazoxanide+Ribavirin	100	Not yet recruiting
NCT04836299	Phase 2	IVM vs Placebo	90	Not yet recruiting
NCT04681053	Phase 3	IVM	80	Recruiting
NCT05231603	Phase 3	IVM vs Placebo	300	Recruiting
NCT04429711	N/A	IVM	100	Recruiting
NCT04723459	N/A	IVM impregnated mask	150	Recruiting
NCT04729140	Phase 4	IVM Tablets vs Doxycycline Tablets vs Placebo	150	Recruiting
NCT04425707	N/A	IVM	100	Recruiting
NCT04834115	Phase 3	IVM Tablets vs Placebo	400	Recruiting
NCT04959786	Phase 2, 3	IVM+ribavirin+nitazoxanide+zinc	100	Recruiting
NCT04373824	N/A	IVM	50	Recruiting
NCT04472585	Phase 1, 2	IVM Injectable Solution vs Injectable Placebo vs Zinc vs Placebo empty capsule vs Oral IVM	180	Recruiting
NCT04447235	Phase 2	IVM vs Placebo vs Losartan	176	Recruiting
NCT04445311	Phase 2, 3	IVM	100	Recruiting
NCT04392713	N/A	IVM Tablets	100	Recruiting
NCT04351347	Phase 2, 3	IVM	300	Recruiting

NCT04435587	Phase 4	IVM vs Combined ART/HCQ	80	Recruiting
NCT04399746	Phase N/A	IVM vs Azithromycinvs Cholecalciferol	30	Recruiting
NCT04885530	Phase 3	IVM vs Fluvoxamine vs Fluticasone vs Placebo	15000	Recruiting
NCT04510194	Phase 3	IVM vs Metformin vs Placebo vs Fluvoxamine	1350	Recruiting
NCT05056883	Phase 3	IVM tablets vs Placebo	1000	Recruiting
NCT04384458	N/A	IVM vs HCQ	400	Recruiting
NCT04951362	Phase 2, 3	IVM nasal spray	117	Recruiting
NCT04703608	Phase 3	IVM vs ASP vs Placebo	1200	Recruiting
NCT05041907	Phase 2	IVM vs Favipiravir vs Monoclonal antibodies vs No treatment vs Remdesivir	750	Recruiting
NCT02735707	Phase 3	IVM vs Other drugs	10000	Recruiting

Tables: Completed: the study ended; Terminated: the study stopped early; Withdrawn: the study stopped at the beginning before the patient's enrollment; Recruiting: the study is still recruiting the patients; Active, not recruiting: the study is ongoing, without enrollment of other patients; Not yet recruiting: the study has not started to recruit patients; N/A: not applicable.
